# Optimizing Denture Success: A Strategy for Prosthodontic Rehabilitation in a Patient With Compromised Alveolar Ridges

**DOI:** 10.7759/cureus.64992

**Published:** 2024-07-20

**Authors:** Pooja M Chitlange, Seema R Kambala, Vedant Pathak, Khushbu Doshi, Mithilesh M Dhamande

**Affiliations:** 1 Department of Prosthodontics and Crown and Bridge, Sharad Pawar Dental College, Datta Meghe Institute of Higher Education and Research, Wardha, IND

**Keywords:** hobkirk technique, complete denture, resorbed alveolar ridge, neutral zone, flabby tissue

## Abstract

The ill-fitting and unstable complete denture prosthesis is the most commonly faced problem by patients with compromised resorbed ridge. The retention, support, and stability of dentures are compromised in severely resorbed ridges, leading to difficulty in mastication and swallowing. The flabby tissue can be managed by using appropriate mucostatic impression techniques, and severely resorbed ridges can be managed by the coordination of a complete denture prosthesis with the neuromuscular function using a neutral zone for the arrangement of artificial teeth. This case report presents a combination of the Hobkirk technique and the neutral zone concept for the rehabilitation of the flabby tissue and severely resorbed alveolar ridge.

## Introduction

Dentures will be more stable if the denture-bearing areas are recorded in a functional state. However, it may be difficult to maintain denture stability when the condition of the denture-supporting areas is inadequate or compromised. Among the compromised residual ridge situations, the most common are flabby ridges, atrophic ridges, and knife-edge ridges [1]. Flabby tissue and severely resorbed residual ridges are the most common findings in elderly edentulous patients. Flabby tissue refers to a physiological condition that occurs due to the presence of a natural tooth in the opposing arch of an edentulous ridge [2]. The residual ridge becomes soft, spongy, and less dense over time due to the resorption of bundle bone and the replacement of bone by hyperplastic connective tissue. This happens due to various reasons, including prolonged use of a faulty denture, trauma, or underlying conditions affecting bone density. The prevalence of flabby tissue is the highest in the maxillary arch (24%), followed by the mandibular arch (5%). Forces of mastication displace the mobile fibrous tissue, leading to instability of the denture and loss of peripheral seal [3]. It can be managed appropriately by modified impression techniques, such as the Hobkirk technique [4], the Zaffurala technique [5], the window tray technique, and the split-tray technique [6].

The rate of resorption in the mandibular ridge is faster than in the maxillary ridge. The stability, retention, and support of a denture prosthesis are compromised in a severely resorbed ridge, leading to difficulty in mastication and swallowing. All oral activities, including talking, chewing, swallowing, grinning, and laughing, depend on the coordinated, highly customized actions of the complete stomatognathic system. The key to a good, successful denture prosthesis is the coordination of the entire denture with the neuromuscular function [6]. The region where the forces acting on the lips, cheeks, and tongue are equivalent is known as the neutral zone. It is the potential area between the tongue from the inside and the lips and cheeks from the outside of the oral cavity. In a highly resorbed mandibular arch, the artificial teeth should be placed in the neutral zone for the complete denture to be successful [7]. Numerous materials, including tissue conditioners, impression compounds, silicone, modeling plastic, wax, and soft liners, have been proposed for recording the neutral zone.

This clinical report describes the rehabilitation of a completely edentulous flabby ridge and a severely resorbed ridge using the Hobkirk technique and the concept of the neutral zone, respectively. The impression for a complete denture prosthesis of the maxillary arch with anterior flabby tissue was made using the Hobkirk technique. For the retention and stability of the lower denture, teeth arrangement was done in the neutral zone region.

## Case presentation

**Figure 1:**
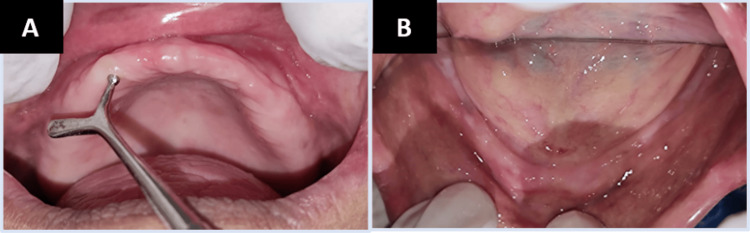
A: Flabby tissue in the maxillary anterior region. B: Severely resorbed mandibular arch.

A 67-year-old female patient reported to the Prosthodontics Department at Sharad Pawar Dental College, Datta Meghe Institute of Higher Education & Research (DMIHER), Sawangi Meghe, Wardha, India, primarily complaining of a fractured complete denture prosthesis. Oral examination revealed fibrous mobile tissue in the anterior region of the maxillary arch (Figure 1A). The mandibular ridge was severely resorbed and well-rounded in shape (Atwood class V) (Figure 1B). All the possible treatment options, such as implant-supported prostheses, implant-supported overdentures, and conventional complete denture prostheses, including pre-prosthetic surgery, were explained to the patient. As the patient was not willing for pre-prosthetic surgery and an implant-supported prosthesis, a conventional complete denture prosthesis was planned with some modifications for flabby tissue and a severely resorbed residual alveolar ridge after obtaining the patient's consent.

**Figure 2:**
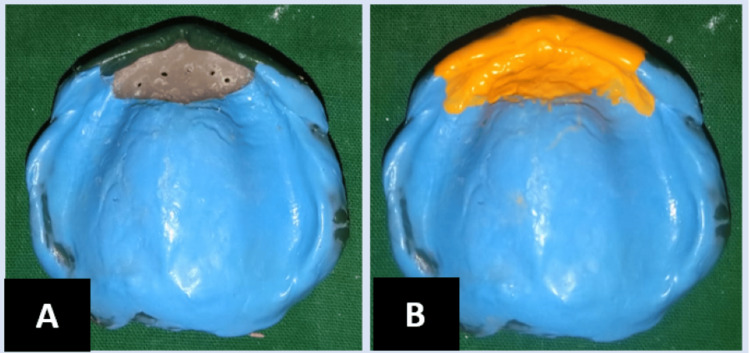
A: Removal of the impression material from the region of flabby tissue. B: Recording of flabby tissue using the Hobkirk technique.

The primary maxillary and mandibular impressions were made using an alginate impression material, and primary impressions were poured to obtain primary casts. For the flabby ridge, a double spacer wax was adapted on the maxillary cast, and a complete single spacer wax was adapted on the mandibular cast. The special trays were fabricated using clear acrylic. The borders were recorded using a green stick impression compound, and the final impression for the maxillary arch was made using a medium-body polyvinyl impression material, and for the mandibular arch, a light-body polyvinyl material was used. The impression material from the flabby tissue region was removed; multiple small relief holes were made for the removal of the excess impression material and recorded again using a light-body elastomeric impression material (Figure 2). Impressions were poured into a type 3 gypsum product; final casts were obtained.

**Figure 3:**
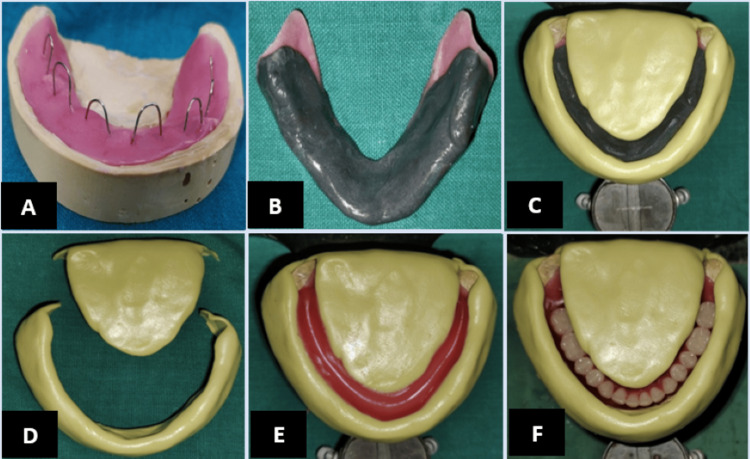
A: Record base with spurs. B: Recording of the neutral zone. C: Putty index. D: Transfer of the putty index. E: Mandibular rim fabrication according to the neutral zone. F: Teeth arrangement according to the recorded neutral zone.

**Figure 4:**
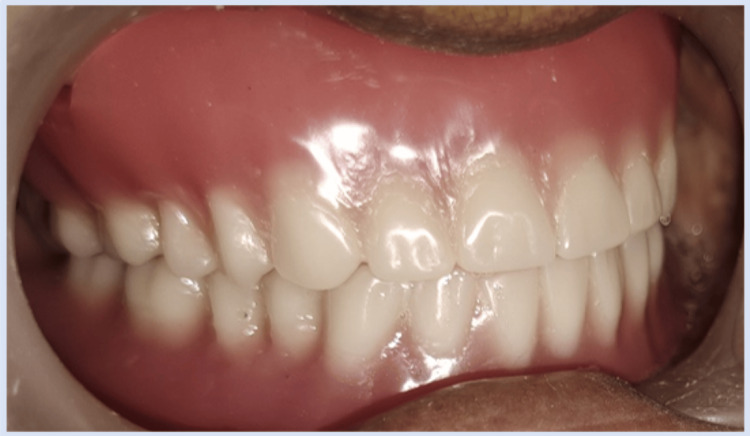
Denture insertion

Record bases and wax rims were fabricated for recording jaw relation. The orientation jaw relation was recorded using a springbow type of facebow, and the recorded jaw relation was mounted on the Hanau Wide-Vue (Whip Mix Hanau™ Wide-Vue, Whip Mix, Kentucky, USA) semi-adjustable articulator. An extra mandibular record base was fabricated and incorporated with spurs of stainless-steel wire, which corresponded to the height of the occlusal rims (Figure 3A). The neutral zone was recorded using the green stick compound by asking the patient to perform various activities, like gulping, licking, sucking, and pronouncing vowels (A, E, I, O, and U) (Figure 3B). The putty index was made using putty consistency elastomeric impression material (Figure 3C), and the putty index was preserved for later use (Figure 3D). A mandibular wax rim was adjusted according to the putty index, which was fabricated according to the recorded neutral zone (Figure 3E). The teeth arrangement was done using the concept of the neutral zone (Figure 3F). After the try-in of the dentures, processing was done by conventional technique. Insertion of a complete denture prosthesis was done (Figure 4). Regular follow-up was done at an interval of 24 hours, seven days, and one month. The patient was comfortable and happy with the prosthesis.

## Discussion

Flabby tissue is a displaceable hyperplastic connective tissue found on the superior aspect of the residual ridge. Kelly et al. (1972) were the first to describe combination syndrome, which is caused by the presence of a natural tooth opposing an edentulous ridge [8]. Results of previous research have shown the prevalence of fibrous tissue varies with arches, i.e., 24% with the maxillary ridge and 5% with the mandibular ridge. Conventional impression techniques tend to compress the flabby tissue during an impression and displace the denture in an uncompressed state [2]. Flabby tissue can be treated surgically or non-surgically. Surgical excision of flabby tissue is not indicated in patients who have poor oral hygiene and are not willing to undergo surgery. Non-surgical techniques include modifications of conventional impression techniques [6]. Out of three primary impression techniques, i.e., pressure, minimal pressure, and selective pressure impression technique, the minimal-pressure method is the most suitable method for recording flabby tissue [2]. Various modified techniques for recording flabby tissue are described in the literature.

This case report presents the Hobkirk technique, in which the final impression is made using a conventional custom tray. In this technique, the border molding is done using green stick material, and the final impression is made using a medium-body elastomeric material. The multiple relief holes are made in the flabby tissue region, and the impression is recorded using a light-body elastomeric impression material [4]. This is an easy and feasible technique to record flabby tissue in a displaced state.

Residual ridge resorption is a complex physiological process that results after tooth extraction. Ridge resorption is maximum after the extraction of teeth until the first year, and then the rate of resorption is slower. Denture retention and stability are very difficult in severe residual ridge resorption, which leads to difficulty in performing routine activities, such as mastication, swallowing, and speaking [9]. There are various impression techniques, like admix, functional, all-green, and elastomeric impression techniques, described in the literature for the management of severely resorbed ridges [10]. The neutral zone has an additional advantage as it records function according to the movements of the oral musculature, which have a balance between the inward forces of the cheeks and lips and the outward forces of the tongue [10]. Materials like tissue conditioners, impression compounds, modeling plastic, elastomeric materials, waxes, dimethyl siloxane filled with calcium silicate, and silicones have been proposed to record neutral zones in the past. In the present case report, a low-fusing impression material was used to record the neutral zone as it is readily available, feasible, and easily moldable, requires less time, has good flow, and has no risk of fracture or distortion.

## Conclusions

Retention, stability, and support are very important for a successful denture prosthesis. Understanding and managing the flabby and resorbed ridge is crucial for achieving successful outcomes in denture fabrication. This case report describes the rehabilitation of compromised edentulous ridges using the Hobkirk technique for flabby tissue and the neutral zone concept for severely resorbed ridges. A comfortable, well-fitting denture can be achieved by the use of appropriate techniques and materials, which restore oral function and improve the quality of life despite the challenges presented by a compromised ridge.
